# Micronutrient powders to combat anemia in young children: does it work?

**DOI:** 10.1186/s12916-017-0867-8

**Published:** 2017-05-11

**Authors:** Frank T. Wieringa

**Affiliations:** UMR-204 Nutripass, Institut de Recherche pour le Développement, IRD/UM/SupAgro, 911 Av d’Agropolis, Montpellier, France

**Keywords:** Anemia, Micronutrient powder, Iron deficiency, Inflammation, Infants

## Abstract

In developing countries, anemia and iron deficiency in early childhood are two highly prevalent public health problems. Providing caregivers with a powder containing multiple vitamins and minerals (also known as micronutrient powder or MNP) as a food supplement is a widely used strategy to combat these problems. However, concerns exist around MNP programs with regards to effectiveness and potential negative impact on diarrheal disease prevalence and gut flora. Teshome et al. (BMC Medicine 15:89, 2017) recently tested a MNP with a new iron formulation, iron-EDTA, which has a potentially higher bioavailability and thus requires a lower iron content. Nevertheless, neither the new formulation nor the standard formulation decreased anemia prevalence as compared to a control MNP without iron. However, in all groups, anemia prevalence was reduced after 30 days of intervention, and iron deficiency prevalence was significantly lower in children receiving iron-EDTA, showing that the new formulation holds promise. More research is needed to verify whether the lower iron content of these MNPs can also reduce the prevalence of associated side effects.

Please see related article: https://bmcmedicine.biomedcentral.com/articles/10.1186/s12916-017-0839-z.

## Background

In their recent article in *BMC Medicine*, Teshome et al. [1] conclude that home iron fortification of complementary foods in young children is insufficiently efficacious. Anemia and micronutrient deficiencies, including iron deficiencies, are highly prevalent in young children in developing countries. Prevalence of anemia in children aged between 6 and 24 months is often well above 50% [2], while the prevalence of iron deficiency is also high in this age group. Importantly, iron deficiency in early childhood has been associated with delayed cognitive development [3], which may be only partially reversible once iron status is restored. It is therefore not surprising that combating anemia and iron deficiency in early childhood is a main priority on the global public health agenda.

Complementary foods with a low micronutrient density are a major cause of the high prevalence of micronutrient deficiencies, including iron deficiency, in early childhood in developing countries. Thus, the simple addition of micronutrients, in the form of a powder (micronutrient powders; MNPs), to complementary foods in the home setting seems a logical step towards increasing micronutrient levels [4]. Nevertheless, an improvement in iron status will only help reduce anemia caused by iron deficiency, while in several developing countries, iron deficiency is a minor contributor to the overall anemia problem [5]. Moreover, MNPs should be seen as a short-term solution, required until low-nutrient dense complementary foods can be replaced with high-quality, nutrient dense complementary foods providing not only micronutrients, but also protein, essential fatty acids, and energy.

Moreover, although MNPs have been shown to reduce overall anemia and iron deficiency prevalence in children, concerns have been raised on increased morbidity, in particular from diarrheal diseases [6] and negative changes in gut flora [7]. These effects seem directly linked to the relatively high content of iron in MNPs, which is necessary due to the relatively low bioavailability of the currently used iron formulation, iron fumarate. Iron fumarate has excellent organoleptic qualities, yet the bioavailability of iron sulfate (the golden standard albeit limited organoleptic qualities) is approximately three-fold higher. A promising alternative formulation, iron-EDTA, has an approximately two-fold higher bioavailability than iron fumarate and good organoleptic qualities. However, daily intake of EDTA has been limited by strict regulations due to potential health concerns [8].

## Discussion

This is the background against which Teshome et al. [1] tested the efficacy of MNPs prepared with iron-EDTA (3 mg daily) versus iron fumarate (12.5 mg daily), given over a short, 30-day intervention. They conclude that both interventions did not improve iron status or hemoglobin concentrations, as compared to ‘placebo’, and question the effectiveness of MNPs to reduce iron deficiency and anemia prevalence in their setting. However, as described by the authors, the study was primarily designed to compare the two different iron formulations. Furthermore, the short duration of the intervention, subject pre-medication, and the MNP treatment for the ‘placebo’ group further impeded testing of the overall efficacy of MNPs.

Thus, the results may not be as unpromising as they seem. First of all, in the study of Teshome et al. [1], the placebo group did not receive a real placebo powder, but rather a MNP that contained 13 vitamins and minerals, including vitamin A and zinc, but no iron. Both vitamin A and zinc deficiency have been associated with anemia [5, 9], and therefore an effect of the ‘placebo’ intervention on anemia prevalence would be expected. Accordingly, the placebo group may be better referred to as a control group. Indeed, anemia prevalence decreased in the control group from 64.3% to 53.3%, an impressive feat given the very short 30-day intervention. In the iron-EDTA group, anemia prevalence decreased even further, from 64 to 43.7%, although this was not significantly different from the control group. As the study was performed in a malaria endemic area, all children received anti-malarial medications at baseline as well as anti-helminth medication. Thus, the observed decrease in anemia prevalence cannot be solely attributed to the MNPs, as pre-medication can also be expected to have improved hemoglobin concentrations [10]. However, iron deficiency prevalence was almost halved in the iron-EDTA group as compared to controls (44.6% vs. 24.5%, *P* < 0.05), suggesting that the MNPs certainly played a role in reducing anemia prevalence.

In an accompanying online supplement, Teshome et al. [1] reported the results of a meta-analysis showing an effect of MNPs on hemoglobin concentrations to be approximately 3.9 g/L, which is not as much as would have been expected. Nevertheless, there was a high heterogeneity between studies, suggesting that other factors also influence the effectiveness of MNPs. Indeed, in their study, the authors identified one such factor, inflammation. Prevalence of sub-clinical infection is high in children living in developing countries, and the associated acute phase response results in higher hepcidin concentrations and lower absorption of iron from the gut. Therefore, children with inflammation respond less to nutritional interventions to improve iron status [11].

## Conclusions

Thus, the real message provided by Teshome et al. [1] should not be that MNPs do not work, but rather that iron-EDTA may improve the effectiveness of MNP programs. The paper confirms that MNPs are not the solution, but can certainly form part of an effective intervention strategy, especially when the MNPs are optimally formulated concerning bioavailability and the inclusion of other micronutrients essential for erythropoiesis. Additional components of such an integrated intervention strategy should be anti-helminth and anti-malaria treatment, and improvements in water, sanitation and hygiene practices. Research is now urgently required to investigate whether the lower dose of iron-EDTA leads to less potential side effects on diarrheal disease and gut flora.

## Abbreviation

MNP: Micronutrient powder
